# BREMi—A New Tool for the Evaluation of UNESCO Biosphere Reserve Management Effectiveness: Case-study in the Arab Man and Biosphere (ArabMAB) Regional Network

**DOI:** 10.1007/s00267-022-01711-x

**Published:** 2022-09-10

**Authors:** Diane A. MATAR, Brandon P. ANTHONY

**Affiliations:** 1 Independent, Palo Alto, CA USA; 2 Department of Environmental Sciences and Policy, Central European University, Vienna, Austria

**Keywords:** Arab MAB, Biosphere reserve, Evaluation, Management effectiveness, Periodic review, Protected area

## Abstract

Scholars and practitioners have been striving to develop straightforward and effective tools to measure protected area management effectiveness (PAME). UNESCO Biosphere Reserves (BR), with their unique functional and zonation schemes are monitored according to their compulsory 10-year Periodic Review (PR), which is useful for UNESCO’s evaluation purposes but lacks comprehensiveness and utility for adaptive management. Based on existing PAME methodologies, we develop and propose the first quantitative tool for the evaluation of BR management effectiveness, that would enhance and complement the currently used qualitative PR report, and serve the rapid evaluation needed for BR managers to monitor, evaluate, and adapt their management approach to achieve the three functions of BRs. The tool consists of 65 indicators, embodied within the 6 elements of the World Commission on Protected Areas Framework. We then tested this tool, named Biosphere Reserve Effectiveness of Management index (BREMi) to evaluate management effectiveness across the Arab Man and the Biosphere Reserve network involving 17 BRs spanning 8 countries of the Middle East and North Africa. BREMi scores ranged from 4.43 to 8.65 (on a scale between 0 and 10), with a mean of 6.31 ± 1.040. All indicators were considered valuable measures of progress by our respondents, as well as by independent experts. We discuss our findings in light of available literature concerning the Arab region and through the conceptual frames of adaptive management and resilience. Finally, we discuss where the BREMi tool would be most useful for BR management authorities in the iterative process of evaluation and adaptive management.

## Introduction

### Protected Areas and Other International Designations as Conservation Strategies

Protected areas (PA) are considered a key global strategy for the conservation of species populations and habitats (Watson et al. 2014). Consequently, their number and spatial extent has been continuously rising, and is currently estimated at 271,791 PAs in 245 countries and territories (UNEP-WCMC and IUCN 2022). In parallel, other models of site protection under international programs with a conservation focus or component have also been flourishing, all of which aim to contribute to the global sustainability agenda (Schaaf and Clamote Rodrigues 2016). Designations under these international programs include: (1) World Heritage Sites estimated at 1154 properties (257 natural or mixed sites) in 167 states (UNESCO WHC 2022); (2) 2438 Ramsar sites covering over 254 million ha in 171 countries (Ramsar Sites Information Service (2022)); (3) UNESCO Global Geoparks estimated at 177 in 44 countries (UNESCO 2022a); and (4) UNESCO Biosphere Reserves (BR) organized into a network of 738 in 134 countries (UNESCO 2022b). These designations often overlap with nationally designated PAs and sometimes each other, creating Multi-Internationally Designated Areas (Schaaf and Clamote Rodrigues 2016). Though the multitude of designations on the same surface of land/sea emphasizes the importance of these sites for their natural and cultural values, their management and evaluation become increasingly complex due to multiple layers of governance and institutional requirements that are not necessarily proactively aligned (Coetzer et al. 2014; Schaaf and Clamote Rodrigues 2016). Here, we focus on BRs, of which the design typically comprises core area/s that overlap with nationally and/or other legally designated PAs (Dudley 2013; UNESCO 2022b).

### Importance and Requirements to Measure PA Management Effectiveness

The worldwide proliferation of PAs and other sites protected under international instruments have not led to the achievement of any of the Convention on Biological Diversity (CBD) Aichi targets, as global indicators reflect a persisting decline in species numbers and habitats ((SCBD) 2020). Lack of PA Management Effectiveness (PAME) has been increasingly highlighted as one of the main reasons behind this failure to halt biodiversity loss (Juffe-Bignoli et al. 2014; Maxwell et al. 2020), reflected by the failure of achieving Aichi Target 11, which suffered in both quantitative and qualitative elements (Gannon et al. 2019).

Since biodiversity outcomes are influenced by several characteristics including the social and economic contexts of PAs, the relationship of PAME with conservation outcomes is not straightforward and PAME assessment tools have continued to be developed in an attempt to more explicitly capture these links (Carranza et al. 2014; Coad et al. 2015; Eklund et al. 2019). Despite these deficiencies, however, recent evidence has consolidated the persistent global importance of PAs as a strategy for conservation by demonstrating significantly higher species richness and abundances inside than outside PAs (Gray et al. 2016). In their global study, Gray and colleagues (2016) also highlighted the very high cost (including opportunity cost) associated with PA expansion, and subsequently emphasized the critical importance of quantifying the effectiveness of PAs to justify their maintenance and expansion. This link between improved management effectiveness and biodiversity outcomes has also been demonstrated by Gill and colleagues (2017) in their global review of marine PAs. However, the current debate as to whether pursue PA expansion or improve management of existing PAs is moot: both approaches must be strategically considered in tandem (Adams et al. 2019).

### Biosphere Reserves and Their Evaluation

The Man and the Biosphere (MAB) program started in the early 1950s and has evolved substantially in coverage and conceptually. Currently, the UNESCO defines BRs broadly as “learning sites for sustainable development” (UNESCO 2022b) that integrate three main functions (used interchangeably with “objectives” throughout this paper): (1) conservation of biodiversity and cultural diversity; (2) economic development that is socio-culturally and environmentally sustainable; and (3) logistic support, underpinning development through research, monitoring, education and training (UNESCO 2022b). Conceptually, the three functions are pursued through BRs’ three main zones (core, buffer, transition), whereby local communities and all interested stakeholders are involved in participatory planning and management (UNESCO 2022b).

As such BRs differ from PAs in their model, structure and governance system. They are therefore not formally considered as a category of the IUCN PA category system but constitute an independent parallel conservation and development strategy overlapping with the PAs recognized by IUCN (see Matar and Anthony 2017). More specifically, BRs most often overlap with legally designated PAs in their core area(s).

At a high level, the UNESCO MAB Secretariat evaluates BRs’ concept implementation, including the implementation of their three functions through a decadal Periodic Review (PR) evaluation process. This process was introduced as a requirement for the World Network of Biosphere Reserves (WNBR) since the 1996 Seville meeting (UNESCO 1996), and involves State agencies, National MAB Committees, an International Coordinating Council, and an International Advisory Committee for BRs in a well laid-out procedure (detailed in Matar and Anthony 2017). However, with exceptions, the PR process has been widely characterized by protracted periods for submission (10 + years), delayed reporting, poor report quality, lacking essential information needed for iterative self-evaluation of management performance, and a low perceived utility by local management teams (Lotze-Campen et al. 2008; Price et al. 2010; Matar and Anthony 2017, 2018).

### Theoretical Underpinnings

#### Resilience theory for complex social-ecological systems

Social-ecological systems are characterized by non-linearity, surprise/shock, alternative stable states and cross-scale dynamics in space and time (Holling and Sundstrom 2015). As part of a joint process of developing a better understanding of, and response to ‘disturbed regional socio-ecological systems’, Holling launched in 1973 his work on adaptive management, which he describes as resilience theory applied to management of social-ecological systems (Holling 1978; Holling and Sundstrom 2015). Resilient systems are dynamic and evolving due to their capacity to learn and self-organize in times of change (Holling and Sundstrom 2015). In that perspective, resilient systems are characterized by high levels of (1) flexibility, (2) learning capacity, and (3) capacity to recover from occasional shocks through ‘creative collapses’ (Holling and Sundstrom 2015). In contrast, when resilience is low, social-ecological systems are rigid, closed, and seeking security rather than opportunity. The BR concept neatly fits the definition of a social-ecological system because its model is centered around the integration of local communities (i.e., social systems), and landscapes with high biodiversity and cultural values, within three zones. As such the MAB program is supporting the creation of resilient social-ecological landscapes.

#### Adaptive management

The conceptual framework of adaptive management emanates from resilience theory. It is described as a process of learning by doing that incorporates research, planning, management actions, monitoring and evaluation of actions, and adaptation in an iterative manner (Van Wilgen and Biggs 2011; Bertzky et al. 2012). Holling (1978) describes adaptive management as an integrated, multidisciplinary and systematic approach to improving management and accommodating change by learning from the outcomes of management policies and practices. Here, we utilize Bormann et al.’s (2007, 187) definition of adaptive management, being *“a systematic and iterative approach for improving resource management by emphasizing learning from management outcomes. Adaptive management is not simply changing management direction in the face of failed policies; rather, it is a planned approach to reliably learning how to improve policies or management practices over time in the face of uncertainty”*. Adaptive management is mostly applied in situations when the scientific knowledge to predict the impact of the application of some management actions is missing, yet not applying any management actions could be deleterious on the ecosystem (Lee 1993; Whelan 2004).

#### Adaptive management applied to protected areas and biosphere reserves

While the concept of adaptive management was first mentioned in the late 1970s and applied for natural resource management of complex ecosystems (Holling 1978; Walters 1986), it was later adopted by conservation experts as a recommended approach to conservation site management and continues to be mentioned as such in new guidelines and reports (Conservation Measures Partnership 2007; Margules and Pressey 2000; Bertzky et al. 2012). Jacobson et al. (2009, 485) cite adaptive management as a “*commonly identified way to address situations in which ecological and social uncertainty exist*”, a situation that particularly holds true in recent times of rapid environmental changes including climate change. Since the nature of social-ecological systems is complex, prone to uncertainties, and not yet fully understood, adaptive management has been recommended as a key approach to effective PA management (Holling 1978; Margules and Pressey 2000; Salafsky et al. 2002; Tucker 2005; Hockings et al. 2015). When applied to conservation planning, adaptive management entails a continuous process of evaluating impacts of conservation management actions in light of specific objectives and making appropriate adjustments in order to adapt management actions to the evaluation results (Margules and Pressey 2000).

In the case of BRs, adaptive management is of increased relevance when considering the dynamic nature of the BR. Continuous changes have been made to the BR concept as the MAB program progressed; these changes have significant implications on its rightful implementation. Moreover, the “*various governance types in place within UNESCO-MAB’s World Network of Biosphere Reserves reflect the range of interpretation of the BR concept*” (Stoll-Kleemann et al. 2008, 6). In that perspective, the omnipresence of change at both the internal programmatic level and the contextual level (global changes such as climate change, urbanization, Covid pandemic, etc.) calls for an adaptive type of governance and management as emphasized after the Seville meeting, which: “*… made a quantum leap in giving increasing emphasis to the ‘M’ of MAB BRs. It affirmed that BRs are ‘more than protected areas’ but rather a ‘pact’ between the local community and society as a whole. Management should be open, evolving and adaptive”* (Bioret et al.^1998^, 3). This relationship between local communities and other stakeholders working together in participatory, co-management arrangements has been demonstrated to create conditions for social learning and favorable outcomes for diverse stakeholders (Stringer et al. 2006).

With this backdrop, we have identified the need for a PAME-like tool for BRs that would (1) evaluate progress toward set objectives and thereby focus on management effectiveness evaluation; and (2) be perceived useful by managing agencies for iterative self-evaluation and adoption of an adaptive management approach (Matar and Anthony 2017, 2018). We outline below the development, testing and reflection of a novel quantitative tool for these purposes.

## Methods

### Development of the BREMi Tool

#### Review of existing PAME tools and selection of a baseline framework

In an effort to develop a novel PAME-like evaluation tool for BRs, we started by reviewing the literature on the wide array of PAME evaluation tools for rapid assessment. These included the most widely used tools: the Management Effectiveness Tracking Tool (METT) (Stolton and Dudley 2016), the Rapid Assessment and Prioritization of Protected Area Management (RAPPAM) (Ervin 2003), the modified Thread Reduction Assessment index (mTRA) (Anthony 2008), and the Common Reporting Format (CRF) framework (Leverington et al. 2010a, 2010b). We also reviewed literature on indicators of success for BRs to identify BR-specific indicators that should be incorporated in our tool. Van Cuong et al. (2017), who based their study on a Delphi approach using expert opinion to assess attributes of success across 90 BRs, list 11 key factors that point to successful outcomes, yet do not develop indicators for these factors that can be tested and utilized on a wider scale. Second, in their systematic review, Ferreira et al. (2018) develop a framework based on 4 categories and 53 sub-categories which shape BR effectiveness. However, ‘Planning’ and ‘Outcome’ elements are omitted, no indicators are articulated, and their framework was left untested. Thus, significant gaps remain with respect to indicator development and testing.

Based on our reviews, we selected the CRF framework (Leverington et al. 2010b; hereafter ‘Global Study’) which is composed of 33 Headline Indicators (HI) as the most relevant and updated framework of indicators to use as a baseline for the development of a new adapted BR evaluation tool. Its utility is particularly advantageous in that it (1) provides the first integrative and unified set of indicators based on a review of >50 different methodologies (including METT, RAPPAM, and mTRA); (2) is relevant to the adaptive management framework which integrates a process of continuous monitoring and evaluation of management performance; (3) is comprehensive as it embodies the six elements of the WCPA framework (context, planning, process, input, output, outcomes), recommended as an underlying framework for all PAME evaluations (Hockings et al. 2006); and (4) is purposeful in that it aims at creating the first standardized set of indicators for PAs, thereby allowing for comparison of results across years for individual PAs, and comparison across PA sites if desired.

#### Adaptation of the selected CRF framework to create the BREMi tool

After selecting the CRF framework as a baseline for developing a BR-specific evaluation tool, we sought to adapt it to the specificities of BRs, encompassing their model, triple zonation scheme, three functions, and factors identified in the literature as priority factors of successful implementation and management of BRs (Stoll-Kleemann 2007). With these criteria in mind, we first revised the 33 headline indicators identified by Leverington et al. (2010b) in their CRF framework as the set of standard indicators across PAME evaluation tools for all categories of PAs. We found that all of them apply to BRs, however we added a 34th headline indicator within ‘Outcomes’ to accommodate the third function of BRs: logistic support for education, research and monitoring, and evaluate progress toward it (Table 1, F.3). We then worked at the level of each of these 34 headline indicators to identify and develop a concise set of underlying indicators (if necessary), based on the same criteria described above, in an iterative process referring to the detailed description of the WCPA elements in Hockings et al. (2006).

**Table 1:** BREMi tool: an adapted list of indicators for BR management effectiveness evaluation

WCPA elements	BREMi indicators
(A, B, C, D. E, F)	
	34 BHIs (bold)
	65 Indicators (*italic*)
**A. CONTEXT**	
**A.1**	**Level of significance (values)**
A.1.1	*Key ecological values are identified and prioritized*
A.1.2	*Key socio-cultural values have been identified and prioritized*
A.1.3	*Potential for sustainable development is identified and prioritized*
A.1.4	*Site value for environmental research, monitoring and education is identified*
**A.2**	**Extent and severity of threats**
A.2.1	*Threats to nominated values are identified and severity evaluated*
**A.3**	**Constraint or support by political and/or civil environment**
A.3.1	*Civil and political contexts are favorable to management success*
A.3.2	*National authorities and leaders are supportive*
A.3.3	*Local community and civil society is supportive*
**B. PLANNING**	
**B.1**	**Protected area gazettal**
B.1.1	*Core area(s) are gazetted (designated by law) nationally*
B.1.2	*Buffer zone(s) are partially or fully gazetted nationally*
**B.2**	**Legislation and policy framework**
B.2.1	*National protected area legislation is inclusive of BRs*
B.2.2	*Land use planning authorities account for the BR*
**B.3**	**Tenure issues**
B.3.1	*Land ownership status and related issues are well known*
B.3.2	*Issues of land tenure are accounted for in planning*
**B.4**	**Marking and security or fencing of boundaries**
B.4.1	*Core area(s) boundaries are known and demarcated (map/signage)*
B.4.2	*Buffer zone(s) boundaries are known and demarcated (map/signage)*
B.4.3	*The Transition zone’s boundary is known*
**B.5**	**Appropriateness of design (for BR functions)**
B.5.1	*Size and zoning are appropriate to the conservation of significant values*
B.5.2	*Size and zoning are adequate to conservation, sustainable development and research*
**B.6**	**Management planning**
B.6.1	*A management plan for the BR site is developed and adequate*
B.6.2	*Resources needed to reach set management objectives are defined*
B.6.3	*Management targets specific to the site values are determined*
B.6.4	*Indicators to monitor progress toward set targets are developed*
B.6.5	*Periodic Review and updating of the management plan is scheduled*
**C. INPUT**	
**C.1**	**Adequacy of staff numbers**
C.1.1	*Staff number is adequate for effective management of the BR*
C.1.2	*Staff is adequately allocated to reach management objectives*
**C.2**	**Adequacy of current funding**
C.2.1	*Funds necessary to reach set management objectives are available*
C.2.2	*Available funds are allocated based on management objectives*
**C.3**	**Security and reliability of funding**
C.3.1	*Funds for the achievement of management objectives are secured*
C.3.2	*Sustainable financing mechanisms are in place*
**C.4**	**Adequacy of infrastructure, equipment and facilities**
C.4.1	*Appropriate vehicles, equipment and facilities are available*
**C.5**	**Adequacy of relevant and available information for management**
C.5.1	*Resources for monitoring set indicators and targets are available*
C.5.2	*Information needed to adequately manage the site is available*
**D. PROCESS**	
**D.1**	**Effectiveness of governance and leadership**
D.1.1	*Governance type of the BR is adequate*
D.1.2	*Governance systems are free from corruption*
D.1.3	*Leadership is effective and adequate*
**D.2**	**Effectiveness of administration including financial management**
D.2.1	*Administrative/financial processes are adequate and effective*
**D.3**	**Management effectiveness evaluation undertaken**
D.3.1	*Management effectiveness evaluation is undertaken*
D.3.2	*Staff meetings are used for learning and adapting*
**D.4**	**Adequacy of building and maintenance systems**
D.4.1	*Maintenance of equipment and infrastructure is adequate*
**D.5**	**Adequacy of staff training**
D.5.1	*Training is adequately provided for staff based on needs*
**D.6**	**Staff/other management partners skill level**
D.6.1	*Expertise and skill level of staff and partners are adequate*
**D.7**	**Adequacy of human resource policies and procedures**
D.7.1	*Management policies and procedures are defined and adequate*
**D.8**	**Adequacy of law enforcement capacity**
D.8.1	*Responsible authorities are capable of enforcing policies and laws inside the BR*
**D.9**	**Involvement of communities and stakeholders**
D.9.1	*Stakeholders are involved in planning and decision-making*
**D.10**	**Communication program**
D.10.1	*Effective means of communication are used with stakeholders*
D.10.2	*An environmental awareness and education program is in place*
**D.11**	**Appropriate program of community benefit/assistance**
D.11.1	*Community use of natural resources is identified*
D.11.2	*Projects and activities of direct community benefit are in place*
**D.12**	**Visitor management (visitors catered for and impacts managed appropriately)**
D.12.1	*Ecotourism visitors are well catered for*
D.12.2	*Visitors’ impacts on values are controlled*
**D.13**	**Natural resource and cultural protection activities undertaken**
D.13.1	*Activities to conserve natural resources are implemented*
D.13.2	*Activities to protect cultural resources are implemented*
**D.14**	**Research and monitoring of natural and cultural management**
D.14.1	*Relevant research on natural and cultural values is undertaken*
D.14.2	*Condition/trends in the state of biodiversity values are monitored*
D.14.3	*Condition/trends in the state of cultural values are monitored*
**D.15**	**Threat monitoring**
D.15.1	*Major threats are monitored and reported*
**E. OUTPUTS**	
**E.1**	**Achievement of set work program**
E.1.1	*Planned targets/objectives are being achieved*
**E.2**	**Results and outputs produced**
E.2.1	*Planned outputs of work program are delivered*
**F. OUTCOMES**	
**F.1**	**Conservation of nominated values**
F.1.1	*Condition of the cultural heritage is well maintained*
F.1.2	*Natural integrity and biodiversity values are well conserved*
F.1.3	*Threats to nominated values are controlled/reduced*
**F.2**	**Effect of BR management on local community**
F.2.1	*The BR socio-economically benefits local community*
**F.3**	**Education, research and monitoring**
F.3.1	*Environmental awareness has increased based on activities*
F.3.2	*The site is regularly used for environmental research and monitoring*

The alphabetical codes assigned to each level (WCPA element, BHI, Indicator) are created to facilitate data analysis as well as in-text referencing

In addition, we purposefully aimed to incorporate the PR review process required by the MAB program into our indicators because of the necessity to conduct and timeously submit the PR review. Further we sought to integrate principles of adaptive management under ‘Planning’ (see Table 1., B.6.5, D.3.1, D.3.2), recognizing the established importance of adopting an adaptive management approach for successful BR management and achievement of desired outcomes (as defined by functions).

This work resulted in a list of 65 indicators embedded in 34 headline indicators, within the 6 elements of the WCPA framework. Generally, we tried to limit the number of indicators to a ‘minimum’ to have a comprehensive assessment adapted to the ArabMAB network (see below), while being mindful of keeping the length as manageable as possible to users. Finally, we independently cross-checked the indicator list with a local BR expert with deep knowledge and experience in the MAB program internationally and regionally, and with a staff member of IUCN North Africa Program who oversees the MAB program regionally. They both provided confirmation of its comprehensiveness and relevance to the Arab region’s context.

Utilized as a tool for quantitative evaluation of BR management effectiveness, we named this adapted list of indicators the Biosphere Reserve Evaluation of Management index (BREMi), detailed in Table 1. Accordingly, the 34 headline indicators were re-labeled BHIs in reference to BREMi HI, in order to distinguish them from those used in the CRF.

### BREMi Evaluation Differentiation from Periodic Review Evaluation

Table 2 provides a summary of the main differences between BR evaluations utilizing the PR Forms (old and newer versions) compared to utilizing the BREMi tool, highlighting its unique complementary value.

**Table 2:** Comparison of PR and BREMi-based evaluations of BR(s)

PR form (2002 version)	PR form (2013 version)	BREMi
Self-evaluation	Self-evaluation	Self-evaluation
Qualitative	Mostly qualitative^a^	Mostly quantitative^a^
Description based	Result/Action based	Result/Action based
BR *concept implementation* focused	BR *concept implementation* focused	*Management effectiveness* focused, integrating BR conceptual aspects
Description of present BR status; i.e., answers the question: *what have you been doing so far?*	Description of present BR status; i.e., answers the question: *what have you been doing so far, including in light of past PRs?*	Assessment of gap toward desired “optimal” BR status; i.e., answers the question: *how far are you from doing your best?*
Built on conceptual definition of BR	Built on conceptual definition of BR	Built on accumulated evidence of success factors for BRs
“Past to present” focus	“Past to present” focus	“Present to future” focus
Evaluation unit is the BR	Evaluation unit is the BR	Evaluation unit is the BR managing organization

^a^Can be complemented with quantitative/qualitative data for explanation/justification

As such, the BREMi tool provides an additional and necessary opportunity for BR managers to run rapid, more frequent assessments of their management quantitatively and complements the PR evaluation process which is qualitative and only takes place every 10 years. It also supplements the range of topics covered by the PR Form (UNESCO 2013) by covering critical aspects of management not directly addressed by the PR process. Examples include “*Civil and political contexts are favorable to management success* (A.3.1)” and “*Governance systems are free from corruption* (D.1.2)” for governance; as well as “*Leadership is effective and adequate* (D.1.3)”, “*Management effectiveness evaluation is undertaken* (D.3.1)”, “*Staff meetings are used for learning and adapting* (D.3.2)” and “*Responsible authorities are capable of enforcing policies and laws inside the BR* (D.8.1)” in management; all of which are proven factors to critically impact BR effectiveness (Stoll-Kleemann 2007).

### Online Survey

#### Survey population

The second phase of our research consisted of testing the novel BREMi tool on a regional network of BRs. We selected the ArabMAB regional network for several reasons: (1) in a previous study we reviewed the effectiveness of the PR process in the ArabMAB and found that it was fraught with challenges (Matar and Anthony 2017), hence the value of testing a different approach and sharing the results with local management authorities; (2) there is a lack of data and documentation on the Arab regional implementation and management of BRs due to difficulty accessing information partly determined by security issues, language barriers, and difficulty in trusting external researchers with local information (Matar 2015); and (3) the first author is culturally connected to the region and has access to the regional network, languages and relations needed for a trustful partnership. We developed a survey for BR senior management staff in the ArabMAB network, selected and validated with the help of UNESCO-MAB offices and the IUCN Mediterranean (IUCN-Med) regional office’s North Africa Program Coordinator.

#### Survey protocol and administration method

The survey protocol consisted of 26 closed and open-ended questions (ranking, scoring, Likert scale, and multiple choice), allowing for the collection of demographic information as well as quantitative and qualitative data (Supplementary Material), in addition to filter questions that aimed at excluding BRs that self-identify as having ‘no operational management’ from conducting the BREMi-based self-evaluation of management.

We selected SurveyMonkey as the online administration method, and pilot tested the draft survey protocol to ensure technical validity, clarity, and appropriateness of length. Our pilot test respondents represented a diverse level of expertise in the subject, including (i) an academic professor in the field of conservation; (ii) a research peer who is not familiar with the topic; and (iii) an employee of an environmental conservation NGO in the Arab region (not participating in the actual survey). They were asked to give feedback on clarity, simplicity, length and flow of the survey, which we used to refine and finalize the protocol. Since language has frequently been identified as one of the main factors that prevent access to information in the Arab region (Matar 2015), we provided the full range of local language options that respondents may be comfortable with (Arabic, French, English). An introductory letter ensuring anonymity and confidentiality (unless stated otherwise by survey respondents) was included in the survey protocol. We sent the final survey to 25 BR management representatives in 11 countries between 2013 and 2014. We had a strong response rate of 88% from 22 of the 25 BRs in the ArabMAB network at the time of the survey. Of the 22, 17 BRs from 8 countries: Algeria, Egypt, Jordan, Lebanon, Sudan, Tunisia, UAE, and Yemen, conducted the BREMi assessment while 5 BRs stated that they have “no operational management” in place.

#### BREMi-based assessment

The BREMi-based evaluation was integrated as a question in the survey protocol and was designed as a list of 65 consecutive indicators i.e., we omitted BHI and WCPA element titles (refer to Table 1) for simplification, however reinstated them in our data entry, analysis and interpretation.

In the Global Study, Leverington et al. (2010b) demonstrated how the CRF framework can be used as a tool for standardized quantitative evaluation of PAME. They created a scoring system with an interval of 0–1 for each indicator, and a categorization system of mean management scores as follows: sound (>0.666); basic (0.501–0.666); basic with major deficiencies (0.333–0.500); clearly inadequate (<0.333). We adopted the same quantitative methods of assessment for the BREMi tool, however we multiplied the ranges by a factor of 10 to make the scoring and calculation process easier. Accordingly, we asked respondents to consider their own BR and allocate a score to all 65 indicators on a scale from 0 to 10, where 0 represents the lowest score (no management/no progress) and 10 represents the best score (excellent management/ideal situation). We later adjusted the scores to the 0–1 scale to allow a rough comparison with the results of the Global Study. In addition to scoring, we asked respondents to assess the relative importance of each indicator to their BR management effectiveness, by assigning a “yes” value for “indicator is relatively important to effective management”, or a “no” value for “the indicator is relatively not important to effective management”.

#### BREMi data analysis

We analyzed quantitative data using IBM^®^ SPSS^®^ Statistics (ver. 20) to conduct univariate and bivariate descriptive statistics, including: measures of dispersion; central tendency; Pearson’s Correlation to explore correlations between interval level variables; and ANOVA to compare means between various groups. We used non-parametric tests including Spearman’s Correlation and Kruskal–Wallis as alternative tests for correlations when data did not meet the assumptions of the parametric tests (i.e., linearity, normal distribution). Alpha was set at 0.05 for all tests.

First, we calculated means of the 34 BHIs from all associated indicator scores, each of the 6 elements of the WCPA framework, and overall BREMi management effectiveness score for each BR. Overall scores were obtained by calculating the averages for the 34 BHIs scores obtained for each BR. Moreover, we calculated country BREMi scores as averages of scores of all participating BRs from each country. We subsequently tested for correlation between BREMi scores and Human Development Index (HDI) values (UNDP 2014), as PA management effectiveness scores were found to be significantly higher in those countries with high and medium HDI ratings (Leverington et al. 2010a). Lastly, we tested for correlation between BREMi scores and two other factors shown to influence management effectiveness, i.e., surface area (Anthony and Shestackova 2015), and year of designation (WWF International 2004).

## Results

### BREMi Scores for The ARAB Biosphere Reserves

**Fig. 1:**
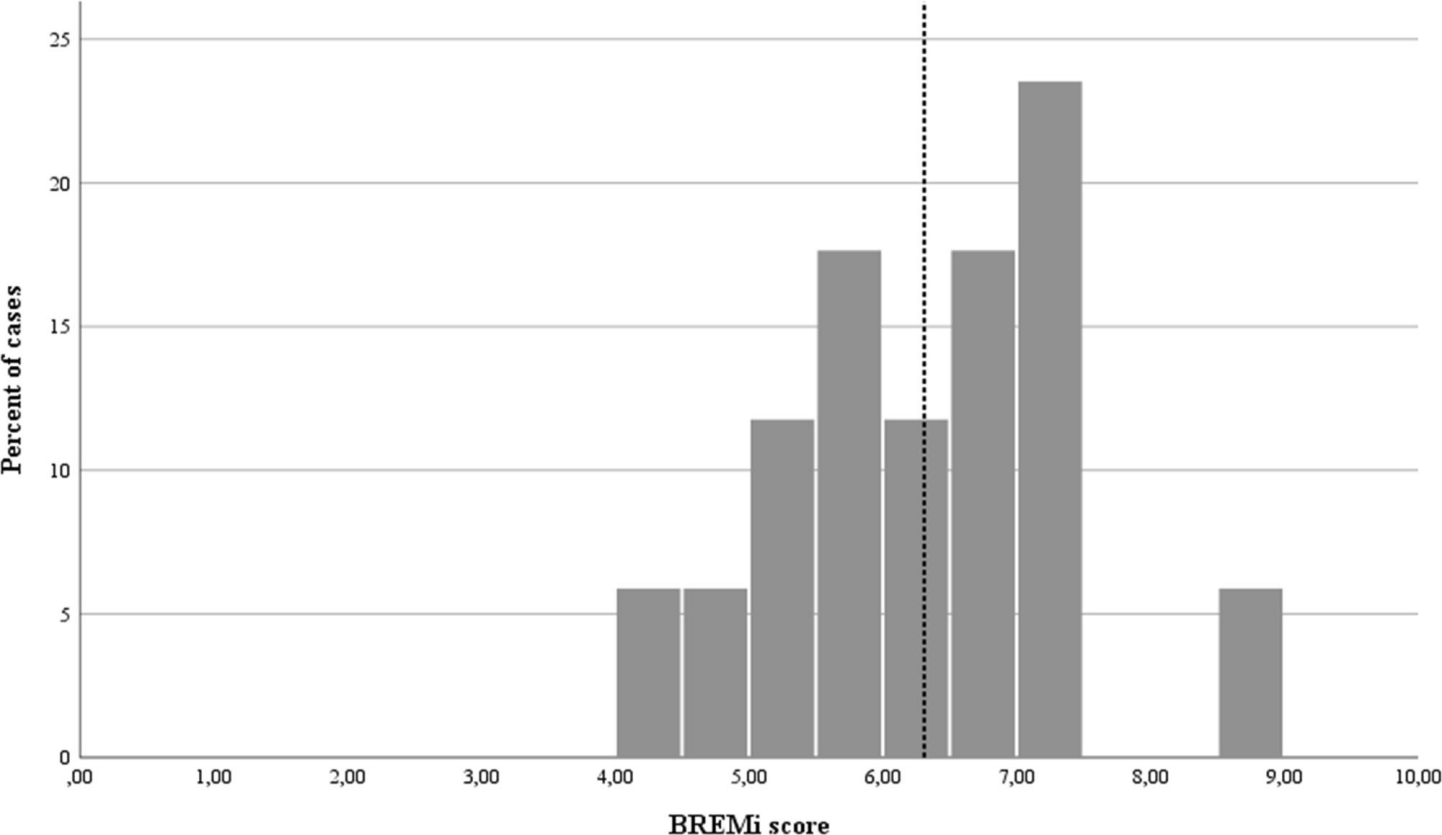
Distribution of mean BREMi scores for BR assessments across the ArabMAB network (*n* = 17). Mean BREMi score (6.31) across all assessments is shown as a dashed vertical line

Overall BREMi scores across the 17 BRs ranged from 4.43 (“basic with major deficiencies”) to 8.65 (“sound”), with a mean of 6.31 ± 1.040 (Fig. 1) falling on the high end of the “basic” management range (5.01–6.66). In total, 35.3% of the Arab BRs scored in the “sound” range, 52.9% in “basic”, 11.8% in “basic with major deficiencies” and 0% in “clearly inadequate”.

### Trends within Countries and Across Contexts

**Fig. 2:**
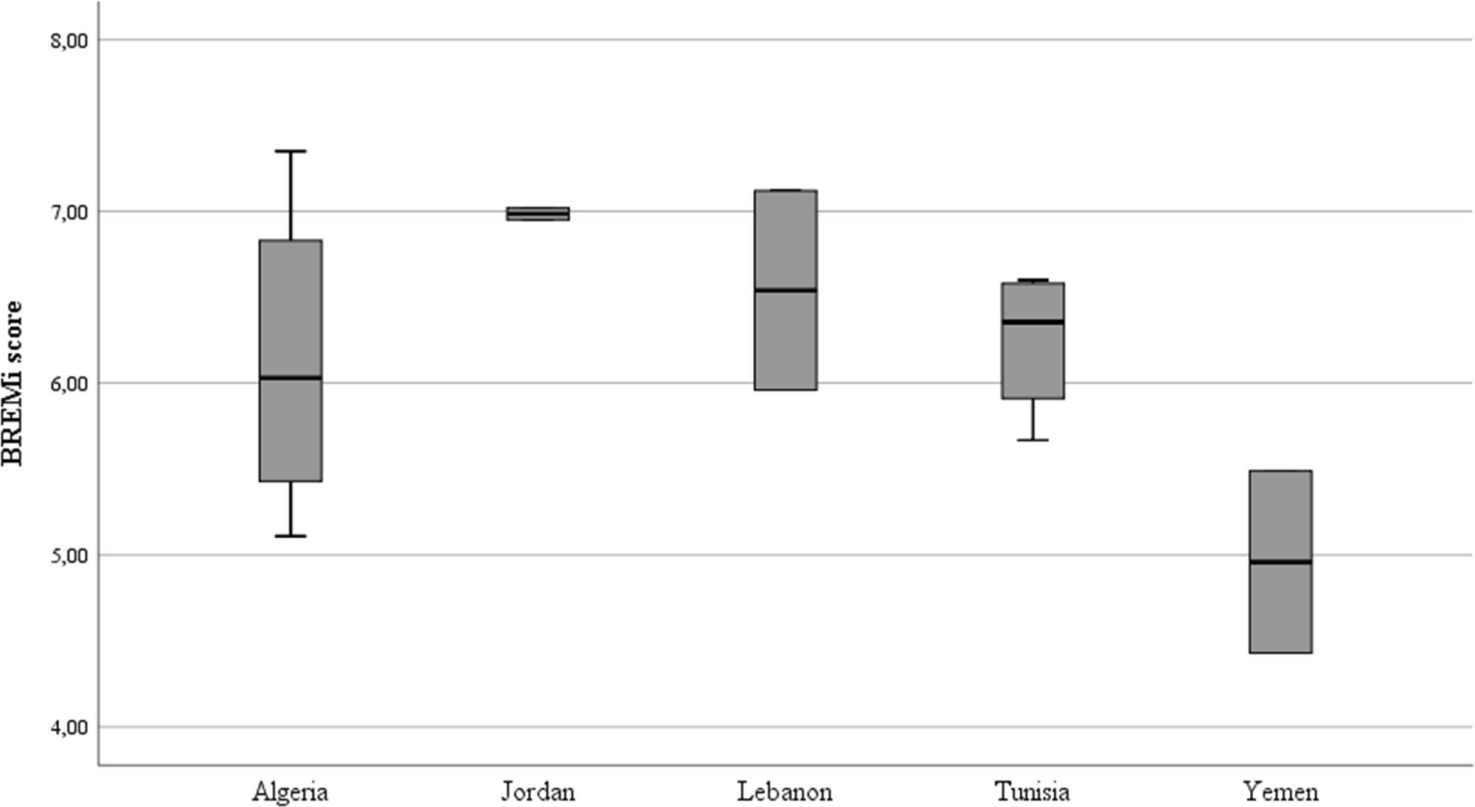
BREMi score per ArabMAB country (three countries where *n* = 1 omitted to respect anonymity and confidentiality)

The Arab BRs did not consistently score in the same range within countries. Only 3 of the 5 assessed countries with more than one BR in the sample had all their national BRs scoring in the same management range, notably Jordanian BRs both scored in the “sound” range, while Tunisian BRs consistently scored in the “basic” range, and Yemeni BRs scored in the “basic with major deficiencies” range (Fig. 2). Differences across geographic or economic contexts are difficult to infer due to the small number of cases (including those where *n* = 1), however some interesting findings are presented below.

Differences in mean country scores were not statistically significant (*p* = 0.168). In addition, individual BR BREMi scores and mean country BREMi scores were non-significant among the four HDI categories set by the United Nations Development Program (*p* = 0.286 and *p* = 0.054, respectively) (UNDP 2020). Furthermore, BREMi scores showed a moderate but non-significant correlation with the corresponding country’s HDI value (Spearman’s *r* = 0.389, *p* = 0.123) suggesting that, contrary to the Global Study, BR management performance in the ArabMAB region is not strongly associated with the level of Human Development of the country. Similarly, BREMi scores were independent of BR area (*p* = 0.242) and year of designation (*p* = 0.403).

When grouped and compared sub-regionally, West Asia (i.e., Jordan, Lebanon, UAE, Yemen) and North Africa (Algeria, Egypt, Sudan, Tunisia) had very similar sub-regional average BREMi scores of 6.30 ± 1.046 and 6.31 ± 1.093, respectively, with no significant difference (*p* = 0.985).

### Trends Across Different Aspects of Management

**Fig. 3:**
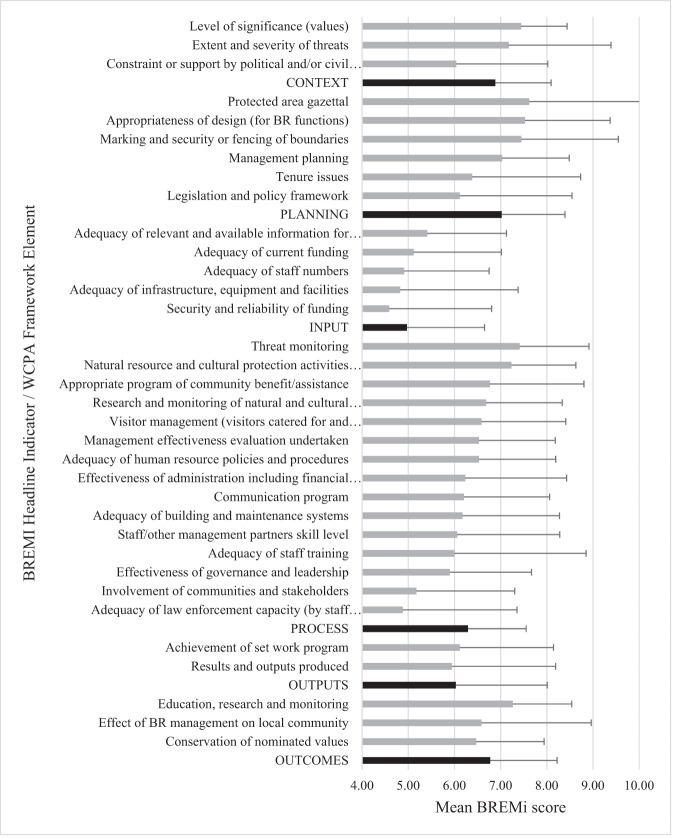
Mean scores and sd for 34 BREMi Headline Indicators (BHIs) (gray bars) and WCPA framework elements (black bars) (*N* = 17)

When mean scores were calculated for each of the elements of the WCPA framework for the Arab BRs regional evaluation, the “planning” element scored highest (7.02 ± 1.372) followed by “context” (6.89 ± 1.208), while the “input” indicator scored the lowest with a mean of 4.97 ± 1.681. Mean scores were calculated for the 34 BHIs, revealing the following notable patterns (Fig. 3):

- 4 of the 6 “planning” BHIs were among the 10 highest scoring BHIs. The lowest scoring planning BHI was *legislation and policy framework*.
- The 10 highest scoring BHIs also included *level of significance (values)* and *extent and severity of threats* from the “context” element of the WCPA framework, *education research and monitoring* from the “outcomes” element, and 3 of 15 “context” BHIs.
- All 5 “input” BHIs scored among the 7 lowest with *adequacy of staff numbers*, *adequacy of infrastructure equipment and facilities*, and *security and reliability of funding* being the most deficient “input” BHIs (score < 5.00).
- All “output” BHIs scored in the “basic” range (score 5.01–6.66).
- None of the BHIs scored in the “clearly inadequate” range (<3.33).

### Perceived Importance of Indicators

Survey respondents were asked to rate each of the 65 indicators of the BREMi tool as “important” or “not important” to the management effectiveness of BRs. There was a 93.2% (sd = 6.1) agreement that the 65 indicators were relatively important across the 17 BRs. Further, based on their ratings, all 22 respondents agreed that none of the indicators were “not important”, reflecting the relevance of all selected indicators to the BR managers and contexts.

### BR Managers’ Feedback on The BREMi Tool

Finally, we solicited optional feedback from our respondents considering the utility of the BREMi tool for BR management effectiveness evaluation. Thirteen of 17 respondents who conducted the BREMi-based assessment submitted feedback, and all were positive in nature. Some noteworthy observations were that the tool was ‘*clear and straightforward*’, ‘*easy to use’*, and that it ‘*tackles real problems the BR faces*’. Further, it ‘*enabled* [the management team] *to do a short and rapid assessment*’ and was considered ‘*a very effective evaluation method for a densely populated biosphere reserve covering a large area*’.

## Discussion

### BREMi Results Reveal Insights on Weaknesses and Strengths of Arab BRs’ Management

The analysis of each indicator’s score across the ArabMAB region allows for a rapid assessment of regional weaknesses in management that may guide remediation actions regionally and/or at the individual site level to improve management effectiveness. Indeed our study revealed that “input” indicators scored lowest among management elements, pointing at resource constraints in terms of funding, staff, infrastructure and equipment as well as information. Other notable weaknesses in management relate to legislative aspects across 4 WCPA elements including *legislation and policy framework* in “planning”, *adequacy of law enforcement capacity* in “process”, and *support by political and/or civil environment* in “context”. Moreover, *involvement of communities and stakeholders* in BR planning and decision-making was one of the lowest scoring “process” BHI(s). We suggest this result to be related to the lack of civil support since local community involvement in BR management decisions is proven to increase the sense of ownership and support to the BRs (Van Cuong et al. 2017). As our BREMi tool is designed to track the ‘if’ and ‘to what degree’ management effectiveness has changed, and not the ‘why’, further investigation is needed to identify the obstacles and find contextually-relevant solutions to these gaps.

In the perspective of better situating the strengths and weaknesses of ArabMAB management effectiveness in the global context, we looked for patterns between the BREMi assessment results and the results obtained in the Global Study and an earlier study in the Levant region (Anthony and Matar 2012). We recognize that the difference in study populations and timeframes between the different studies don’t allow for direct comparison, however the use of the same method grounded in the CRF framework supports the identification of similarities. We first found that “planning” is consistently the most effective aspect of management with the three highest scoring indicators being all “planning” indicators in regional and global results. Therefore, “planning” tends to be a strength of management for BRs and PAs internationally. In addition, the three studies reveal a trend in the lowest scoring indicators being consistently related to “input”, indicating that resource constraints are perceived as the biggest challenges to management effectiveness for both BRs and PAs at regional and international scales. In contrast, we noted that *adequacy of law enforcement capacity* (“process”), scored low in both the ArabMAB region and within the Levant (Anthony and Matar 2012), but received a better rating in the Global Study.

Similarities and differences in these observations are important pointers to the scales of the challenges faced by BRs (local vs. global) and can support decision-making for solutions at appropriate scales of governance. For example, reasons for the more region-specific legislative challenges may be related to the political instability and conflicts in the Arab region (Lebanon, Tunisia, Sudan) and the lack of legislative status to the MAB program in several countries in the region (Matar 2015).

### BREMi Tool Fulfills the Need for A Management-focused Evaluation tool

“Monitoring and evaluation for adaptive management” is in the top 5 of 27 influencing factors of BR success according to 204 BR managers globally (Stoll-Kleemann and Welp 2008). Therefore, improving its implementation and effectiveness would significantly contribute to the success of BR management regionally and beyond. However, the PR process of evaluation of UNESCO BRs has proven to be insufficient especially for management effectiveness evaluation, as it is designed to focus on assessing the gap between BR concept and implementation rather than management effectiveness. Recent updates in the PR Form (2013 version) address changes in concept since the beginning of the program and put more emphasis on management and coordination; however, updates didn’t alter the PR tool’s overall purpose. In that perspective, the BREMi, with its scoring system, is a more appropriate tool for appraising management effectiveness since it is designed with a management-focus, and integrates the BR management functions (conservation, sustainable development, and logistic support) as the standard functions and desired outcomes of BR management. If used in combination, the PR and BREMi tools would complement each other for a better evaluation of both concept implementation and management effectiveness.

The ArabMAB experience with PRs and BREMi evaluations provides the first example of the different benefits of using both tools. Based on Matar and Anthony (2017), the PR process in the Arab region was characterized by long periods of submission delay, variable quality of reports that were poor in certain cases, and a low level of understanding or valuing its purpose. In summary, it did not prove to be effective enough in the Arab region to date, and little is known about its effectiveness in specific regions and countries elsewhere. The underlying reasons for poor compliance need to be further defined and addressed. A limited number of studies have identified potential reasons for the lack of effectiveness of the PR evaluation process (Price 2002; Price et al. 2010; Reed and Egunyu 2013; Matar and Anthony 2017), as follows: (1) low level of understanding and/or appreciation of the purpose of the PR, (2) financial limitations and shortage of expertise, and (3) lack of perception and adoption of the PR process as a self-serving learning tool and opportunity by the BR management stakeholders locally. In contrast, the positive feedback received by respondents concerning the BREMi tool shows promising results for adoption of the tool by BR managers since it was perceived as useful and easy to use. Therefore, we recommend the adoption and use of the BREMi tool in parallel to the PR form—in order to remediate some of the gaps of the PR evaluation and improve monitoring and evaluation of BRs in the ArabMAB region, and internationally.

### Using The BREMi Tool for Evaluation and Learning as Part of Adaptive Management

**Fig. 4:**
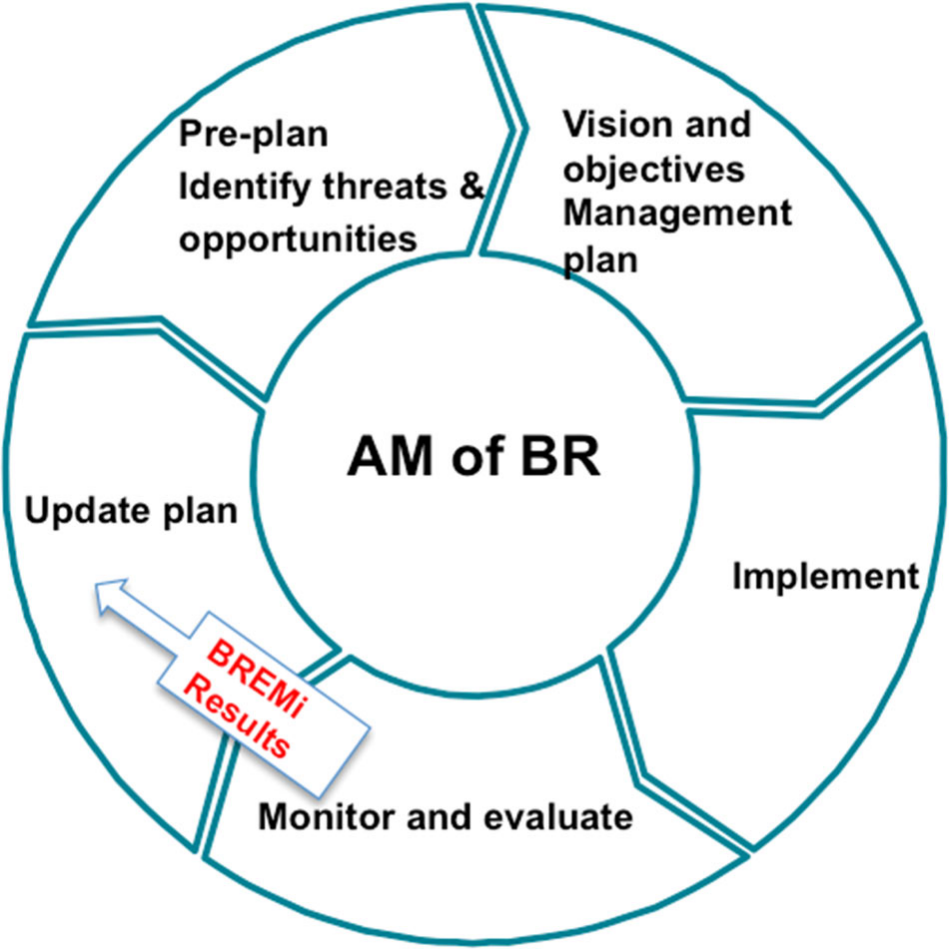
BREMi-based evaluation as part of the adaptive management cycle of BR(s). The cycle represents the first of an iterative process; In subsequent cycles the first 2 steps i.e., “Pre-plan, Identify threats and opportunities” and “Vision and objectives, Management plan” become “Review” and “Revisions to objectives/management plan”

Used as a tool for evaluation and learning, the BREMi tool fits into the adaptive management approach to BR management by allowing for reflection on the usefulness of certain management decisions and policies and subsequent adaptation of plans and processes in an iterative manner (Fig. 4).

As shown in Fig. 4, the evaluation results will provide input and knowledge to review original decisions, test assumptions and make changes in plans and actions accordingly as needed (Margules and Pressey 2000; Salafsky et al. 2001; Salafsky et al. 2002). Moreover, as stipulated in the adopted definition of adaptive management (Bormann et al. 2007), the development and selection of indicators (i.e., BREMi) starts at the pre-planning phase. Hence, the BREMi tool should be adopted by BR managers from the phase where they should consider necessary adaptation of the tool to their BR’s context and management needs i.e., (1) adding or removing indicators based on their contextual relevance, and (2) weighting each of the BHIs based on its degree of importance to BR management in its specific context (Anthony and Shestackova 2015; Hockings et al. 2015). Thus, the BREMi tool is also useful as a planning tool as it allows for:

1. Objective and target setting and alignment with indicators.
2. Planning and budgeting for evaluation processes and participatory mechanisms.
3. Planning for alternative scenarios and for related policy changes in case of failure (Swanson and Bhadwal 2009).
4. Assessing research and knowledge needs and planning for their access.
5. Identifying and developing policies that create the enabling environment to fulfill objectives, including incentives to BR managers (Armitage et al. 2009).

The “evaluation for learning and management” phase is an integral part of the adaptive management process. Its implementation is recommended as part of a collaborative and adaptive management approach, where management effectiveness evaluation is conducted using a participatory process fostering discussion around BR management challenges, deliberation and consensus on processes and management decisions. As defined by Selin and Chavez (1995) collaboration involves (1) joint decision-making, (2) power sharing, and (3) collective responsibility of stakeholders for their actions and subsequent outcomes, which also means “risk sharing” of prospective failed policies or outcomes (Armitage et al. 2009). Hence, participatory mechanisms at any stage of the adaptive management cycle, including the ones involving the BREMi tool, can increase ownership of the overall MAB program implementation in the Arab region. Thus, involving local community stakeholders in decision-making about indicators, objectives, outcomes to be monitored, etc. (Anthony and Swemmer 2015), is also part of the solution to improving management effectiveness evaluation in the ArabMAB region. Inclusive and communal institutional arrangements need to be planned and put in place for a successful and comprehensive participatory evaluation, which in turn needs to be planned in advance i.e., integrated in the institutional design and allocated a budget.

### Study Limitations

By using the CRF framework for developing the BREMi tool, we recognize that the inherent limitations of many PAME evaluation tools apply to the BREMi tool as well. Since respondent scoring is utilized to ascertain data on management effectiveness, our study is limited by the subjectivity of our respondents (Cook and Hockings 2011). We have made every attempt to collect data from those respondents whom we believed had the best knowledge of the management indicators we were assessing, however with the lack of published information in the region this is a factor which we could not control for, and which may be liable to overstating or understating performance by the individual assessors (Burgman 2001).

In addition, we recognize that the BR concept and governing documents have evolved since our study was conducted (2014) and therefore some important aspects of management may be deemed relevant and necessary to add to the BREMi tool. This includes the increasing role that BRs have in mitigating and adapting to climate change (UNESCO 2017). However, the BHIs remain largely applicable at this time, especially as the three main functions of BRs defined by the UNESCO MAB Secretariat remain unchanged (UNESCO 2022b). We recommend adapting the language and number of indicators within each BHIs to accommodate specificities of each BR and its context, in addition to changes in the overall conceptual and strategic directions of the MAB program through time. This will allow more nuanced tracking of individual BR management effectiveness through successive assessments.

## Conclusion

The BREMi tool, adapted from the CRF framework constitutes the first standardized set of indicators for rapid evaluation of UNESCO BRs. In our study, we have provided a case-study of its use, and acceptability, in a regional network of the WNBR, the ArabMAB, and demonstrated how the tool can be utilized by local BR governance authorities for a rapid evaluation of their management effectiveness, revealing weaknesses, strengths and gaps in their management at different points in time and through time. The tool would be most useful if integrated in BR management and evaluation plans and used iteratively to assess progress as part of adaptive management. In addition, the standardization of the BREMi’s BHIs and scoring methods provide an opportunity for comparison across BRs nationally, regionally or globally, if adopted and utilized in similar ways. The aim of comparison would be to provide transparency in reporting on progress and an opportunity to track changes through time to learn and exchange lessons on what has worked best in different contexts and across the network of BRs. Finally, we recommend considering BREMi as an additional and valuable tool in the toolbox of methods for evaluation of BRs, complementary to the current qualitative official PR reporting system, and other more outcome-focused tools for the evaluation of the different functions of biosphere reserves (e.g., ecological monitoring tools for the conservation function).

## Supplementary information

Supplementary information

## Data Availability

Due to confidentiality and anonymity agreements, questionnaire data rests with the first author.
