# Supplementary material for: BREMi—A New Tool for the Evaluation of UNESCO Biosphere Reserve Management Effectiveness: Case-study in the Arab Man and Biosphere (ArabMAB) Regional Network
Source: Environ Manage. 2022 Sep 10;70(5):730–45. doi: 10.1007/s00267-022-01711-x (PMC9519677; doi:10.1007/s00267-022-01711-x)
Supplement: Supplementary file 1 — Supplementary information [file 267_2022_1711_MOESM1_ESM.docx]

**UNESCO Biosphere Reserves Management Survey for the Arab Region (English version)**

**(*Note: only relevant questions included for this paper*)**

## Background

### 1) About the Biosphere Reserve (BR)

BR official name: ____________________________________________

#### Country

( ) Algeria

( ) Egypt

( ) Jordan

( ) Lebanon

( ) Morocco

( ) Tunisia

( ) Qatar

( ) Sudan

( ) Syria

( ) UAE

( ) Yemen

## Management Evaluation

#### 21) You are about to start the self-assessment of your BR management effectiveness performance. This is a very important part of the survey, the results of which will be shared with you to help orient your management priorities. Please take 10-15 more minutes to complete this part in consultation with other team members.

#### In the table below, you will find a list of statements that are indicators for management evaluations. For each indicator, please indicate its importance relative to your BR management effectiveness. Then, on the same row, assign a score over 10 reflecting your performance on that same indicator.

**Importance rating**Yes  = the indicator is relatively important to effective management
No  = the indicator is relatively not important to effective management
 **Performance rating scores can range from 0 to 10, where**0   = no progress
5   = average progress
10 = ideal situation achieved

**PLEASE MAKE SURE THAT 2 ANSWERS ARE TICKED ON EACH ROW: ONE FOR “IMPORTANT” AND ANOTHER FOR “PERFORMANCE”.**

| **Indicator** | **Important** | | **Performance rating (score)** | | | | | | | | | | |
| --- | --- | --- | --- | --- | --- | --- | --- | --- | --- | --- | --- | --- | --- |
|  | **Yes** | **No** | **0** | **1** | **2** | **3** | **4** | **5** | **6** | **7** | **8** | **9** | **10** |
| Key ecological values are identified and prioritized |  |  |  |  |  |  |  |  |  |  |  |  |  |
| Key cultural values are identified and prioritized |  |  |  |  |  |  |  |  |  |  |  |  |  |
| Potential for sustainable development is identified and prioritized |  |  |  |  |  |  |  |  |  |  |  |  |  |
| Site value for env. research, monitoring and education is identified |  |  |  |  |  |  |  |  |  |  |  |  |  |
| Threats to nominated values are identified and severity evaluated |  |  |  |  |  |  |  |  |  |  |  |  |  |
| Civil and political contexts are favorable to management success |  |  |  |  |  |  |  |  |  |  |  |  |  |
| National authorities and leaders are supportive |  |  |  |  |  |  |  |  |  |  |  |  |  |
| Local community and civil society is supportive |  |  |  |  |  |  |  |  |  |  |  |  |  |
| Core zone(s) are gazetted (designated by law) nationally |  |  |  |  |  |  |  |  |  |  |  |  |  |
| Buffer zone(s) are partially or fully gazetted nationally |  |  |  |  |  |  |  |  |  |  |  |  |  |
| National protected area legislation is inclusive of BRs |  |  |  |  |  |  |  |  |  |  |  |  |  |
| Land use planning authorities account for the BR |  |  |  |  |  |  |  |  |  |  |  |  |  |
| Land ownership status and related issues are well known |  |  |  |  |  |  |  |  |  |  |  |  |  |
| Issues of land tenure are accounted for in planning |  |  |  |  |  |  |  |  |  |  |  |  |  |
| Core zone(s) boundaries are known and demarcated (map, signage) |  |  |  |  |  |  |  |  |  |  |  |  |  |
| Buffer zone(s) boundaries are known and demarcated (map, signage) |  |  |  |  |  |  |  |  |  |  |  |  |  |
| Transition zone boundary is known |  |  |  |  |  |  |  |  |  |  |  |  |  |
| Size and zoning are appropriate to the conservation of significant values |  |  |  |  |  |  |  |  |  |  |  |  |  |
| Size and zoning are adequate to conservation, development & research |  |  |  |  |  |  |  |  |  |  |  |  |  |
| A Management Plan for the BR site is developed and adequate |  |  |  |  |  |  |  |  |  |  |  |  |  |
| Resources needed to reach set management objectives are defined |  |  |  |  |  |  |  |  |  |  |  |  |  |

| **Indicator** | **Important** | | **Performance rating (score)** | | | | | | | | | | |
| --- | --- | --- | --- | --- | --- | --- | --- | --- | --- | --- | --- | --- | --- |
|  | **Yes** | **No** | **0** | **1** | **2** | **3** | **4** | **5** | **6** | **7** | **8** | **9** | **10** |
| Management targets specific to the site values are determined |  |  |  |  |  |  |  |  |  |  |  |  |  |
| Indicators to monitor progress towards set targets are developed |  |  |  |  |  |  |  |  |  |  |  |  |  |
| Periodic review and updating of the Management Plan is scheduled |  |  |  |  |  |  |  |  |  |  |  |  |  |
| Staff number is adequate for effective management of the BR |  |  |  |  |  |  |  |  |  |  |  |  |  |
| Staff is adequately allocated to reach management objectives |  |  |  |  |  |  |  |  |  |  |  |  |  |
| Funds necessary to reach set management objectives are available |  |  |  |  |  |  |  |  |  |  |  |  |  |
| Available funds are allocated based on management objectives |  |  |  |  |  |  |  |  |  |  |  |  |  |
| Funds for the achievement of management objectives are secured |  |  |  |  |  |  |  |  |  |  |  |  |  |
| Sustainable financing mechanisms are in place |  |  |  |  |  |  |  |  |  |  |  |  |  |
| Appropriate vehicles, equipment and facilities are available |  |  |  |  |  |  |  |  |  |  |  |  |  |
| Resources for monitoring set indicators and targets are available |  |  |  |  |  |  |  |  |  |  |  |  |  |
| Information needed to adequately manage the site is available |  |  |  |  |  |  |  |  |  |  |  |  |  |
| Governance type of the BR is adequate |  |  |  |  |  |  |  |  |  |  |  |  |  |
| Governance systems are free from corruption |  |  |  |  |  |  |  |  |  |  |  |  |  |
| Leadership is effective and adequate |  |  |  |  |  |  |  |  |  |  |  |  |  |
| Administrative/financial processes are adequate and effective |  |  |  |  |  |  |  |  |  |  |  |  |  |
| Management effectiveness evaluation is undertaken |  |  |  |  |  |  |  |  |  |  |  |  |  |
| Staff meetings are used for learning and adapting |  |  |  |  |  |  |  |  |  |  |  |  |  |
| Maintenance of equipment and infrastructure is adequate |  |  |  |  |  |  |  |  |  |  |  |  |  |
| Training is adequately provided for staff based on needs |  |  |  |  |  |  |  |  |  |  |  |  |  |
| Expertise and skill level of staff and partners are adequate |  |  |  |  |  |  |  |  |  |  |  |  |  |

| **Indicator** | **Important** | | **Performance rating (score)** | | | | | | | | | | |
| --- | --- | --- | --- | --- | --- | --- | --- | --- | --- | --- | --- | --- | --- |
|  | **Yes** | **No** | **0** | **1** | **2** | **3** | **4** | **5** | **6** | **7** | **8** | **9** | **10** |
| Management policies and procedures are defined and adequate |  |  |  |  |  |  |  |  |  |  |  |  |  |
| Staff is capable of enforcing policies and laws inside the BR |  |  |  |  |  |  |  |  |  |  |  |  |  |
| Stakeholders are involved in planning and decision-making |  |  |  |  |  |  |  |  |  |  |  |  |  |
| Effective means of communication are used with stakeholders |  |  |  |  |  |  |  |  |  |  |  |  |  |
| An env. awareness and education program is in place |  |  |  |  |  |  |  |  |  |  |  |  |  |
| Community use of natural resources is identified |  |  |  |  |  |  |  |  |  |  |  |  |  |
| Projects and activities of direct community benefit are in place |  |  |  |  |  |  |  |  |  |  |  |  |  |
| Ecotourism visitors are well catered for |  |  |  |  |  |  |  |  |  |  |  |  |  |
| Visitors' impacts on values are controlled |  |  |  |  |  |  |  |  |  |  |  |  |  |
| Activities to conserve natural resources are implemented |  |  |  |  |  |  |  |  |  |  |  |  |  |
| Activities to protect cultural resources are implemented |  |  |  |  |  |  |  |  |  |  |  |  |  |
| Relevant research on natural and cultural values is undertaken |  |  |  |  |  |  |  |  |  |  |  |  |  |
| Condition/trends in the state of biodiversity values are monitored |  |  |  |  |  |  |  |  |  |  |  |  |  |
| Condition/trends in state of cultural values are monitored |  |  |  |  |  |  |  |  |  |  |  |  |  |
| Major threats are monitored and reported |  |  |  |  |  |  |  |  |  |  |  |  |  |
| Planned targets/objectives are being achieved |  |  |  |  |  |  |  |  |  |  |  |  |  |
| Planned outputs of work program are delivered |  |  |  |  |  |  |  |  |  |  |  |  |  |
| Condition of the cultural heritage is well maintained |  |  |  |  |  |  |  |  |  |  |  |  |  |
| Natural integrity and biodiversity values are well conserved |  |  |  |  |  |  |  |  |  |  |  |  |  |
| Threats to nominated values are controlled/reduced |  |  |  |  |  |  |  |  |  |  |  |  |  |
| The BR socio-economically benefits local community |  |  |  |  |  |  |  |  |  |  |  |  |  |
| Env. awareness has increased based on activities |  |  |  |  |  |  |  |  |  |  |  |  |  |
| The site is regularly used for env. research and monitoring |  |  |  |  |  |  |  |  |  |  |  |  |  |

## Feedback

### 22) Please use the space below to give your feedback about the questionnaire and evaluation method, your opinion is highly appreciated (optional).

________________________________________________________________________________________

________________________________________________________________________________________

________________________________________________________________________________________

## Thank You!

### Thank you for taking this survey! Your input is highly appreciated and of added value to UNESCO Biosphere Reserves and conservation in the region.
